# A retrospective study of CT-guided percutaneous irreversible electroporation (IRE) ablation: clinical efficacy and safety

**DOI:** 10.1186/s12885-021-07820-w

**Published:** 2021-02-05

**Authors:** Ziyin Wang, Jian Lu, Wei Huang, Zhiyuan Wu, Ju Gong, Qingbing Wang, Qin Liu, Cangyi Wang, Yu Zhu, Xiaoyi Ding, Zhongmin Wang

**Affiliations:** 1 Department of Radiology, Ruijin Hospital, School of Medicine, Shanghai Jiao Tong University, 197#, Rui Jin Er Road, Shanghai, 200025 People’s Republic of China; 2 Department of Radiology, Ruijin Hospital Luwan Branch, School of Medicine, Shanghai Jiao Tong University, 149#, South Chongqing Road, Shanghai, 200020 People’s Republic of China

**Keywords:** Carcinoma, Renal cell, Ablation techniques, Computed tomography

## Abstract

**Background:**

To evaluate the clinical efficacy and safety of ablating renal cell carcinoma (RCC) by irreversible electroporation (IRE).

**Methods:**

Fifteen patients (19 lesions) with RCC who underwent IRE were retrospectively reviewed. Seven patients had solitary kidneys. Two lesions were located in the renal hilus. One patient had chronic renal insufficiency. Percutaneous biopsy for histopathology was performed. The best puncture path plan was evaluated before CT-guided IRE. The estimated glomerular filtration rate (eGFR) was compared vs baseline at 1–2 months after the ablation. Contrast-enhanced computed tomography imaging changes were evaluated immediately after IRE. Contrast-enhanced computed tomography/magnetic resonance was performed 1 month, 3 months, 6 months, 12 months and every year thereafter. The complications after treatment were also reviewed.

**Results:**

The success rate of the procedure was 100%. The median tumor size was 2.4 (IQR 1.3–2.9) cm, with an median score of 6 (IQR 5.5–8) per R.E.N.A.L. criteria (radius, exophytic/endophytic, nearness to collecting system or sinus, anterior/posterior, and location relative to polar lines). Two cases (3 lesions) were punctured through the liver. In other cases, puncture was performed through the perirenal space. There were no severecomplications in interventional therapy. Transient gross hematuria occurred in 2 patients (centrally located). Self-limiting perinephric hematomas occurred in 1 patient. Needle puncture path metastasis was found in 1 patient 2.5 years after IRE. The subcutaneous metastasis was surgically removed, and there was no evidence of recurrence. There was no significant change in eGFR levels in terms of short- term clinical outcomes (*t* = 0.348, *P* = 0.733). At 6 months, all 15 patients with imaging studies available had no evidence of recurrence. At 1 year, 1 patient (1 of 15) was noted to have experienced needle tract metastasis and accepted salvage radiofrequency ablation (RFA) therapy.

**Conclusions:**

IRE appears to be a safe and effective treatment for RCC that may offer a tissue-sparing method and complete ablation as an alternative therapy for RCC.

## Background

Renal cell carcinoma (RCC) now has the second-highest incidence rate among all malignant tumors of the urinary system. With the evolution and popularity ofinterventional treatment technology, the European Association of Urology (EUA) has recommendedablative therapies for completely destroying tumor tissue and minimizing the injury of normal tissues in the guidelines for the treatment of RCC [1]. Traditional thermal ablation modalities such as radiofrequency ablation (RFA) and microwave ablation (MWA) use extreme temperatures to destroy target tissue. Therefore, thermal collateral damage to adjacent structures and heterogeneous ablation zones due to the heatsink effect are shortcomings. Irreversible electroporation (IRE) is a minimally invasive tumor ablative technique that has emerged in recent years. Although the precise mechanism by which IRE causes cell death remains to be elucidated, its theoretical advantage lies in its ability to induce apoptosis without thermal energy [2–5]. IRE is thought to spare surrounding structures such as blood vessels, connective tissue, and nerves [2]. Therefore, IRE represents an interesting option for the nephron-sparing treatment of renal tumors, even for those with an unfavorable anatomic location (e.g., centrally located and close to the renal pelvis and/or the large hilar vessels). The purpose of the present study was to evaluate the clinical efficacy and safety of RCC to provide a new treatment modality for renal cell carcinoma even in unfavorable anatomic locations and patients with renal insufficiency.

## Methods

This retrospective study was approved by the institutional review board. There was no financial or industry support obtained.

### Patients

From February 2017 to August 2019, 15 patients with 19 RCC lesions treated with IRE were included in the data analysis (Table 1 and Table 2).

**Table 1:** Each Patient Characteristics

Patient No.	R.E.N.A.L nephrometry score	eGRF (mL/min)(preoperative)	eGRF (mL/min)(1-2 M after operative)	Haemoglobin(g/l)(preoperative)	Haemoglobin(g/l)(3-7D after operative)	Operation- related complications	Maximal tumordiameter (cm)	Tumor location	Number of electrodes	Follow-up (month)	Survival
1	7P	98.6	86.1	118	112	None	1.3	R- upper	2	32	Alive
2	8P	44.2	39.2	114	119	None	2.3	R- middle	4	43	Alive
3	9P	32.0	50.1	121	128	None	3.6	L-upper	4	42	Alive
	4P					None	4.2	L-lower	4		
4	8P	19.8	16.2	122	126	None	2.8	L-upper	4	41	Alive
5	6A	31.8	61.4	128	125	None	2.2	L-middle	3	36	Alive
6	6A	48.1	/	118	115	None	1.1	L-lower	2	33	Alive
7	5A	114.4	111.8	141	135	None	2.1	L- upper	4	32	Alive
8	5A	55.4	36.2	119	129	None	1.7	R-upper	3	30	Alive
9	6A	73.3	64.9	121	118	None	3.0	R-upper	4	28	Alive
10	5A	12.93	9.74	109	110	None	2.5	R-lower	3	24	Alive
	9A					perinephric hematomas	4.5	L-lower	4		
11	4A	37.8	43.6	129	120	Transient mild macroscopic hematuria	1.4	R-upper	3	14	Alive
	8 Ah						2.0	R-central	3		
12	8P	31.1	36	118	112	None	4.3	R-lower	4	12	Alive
13	6A	49.2	44	122	131	None	1.3	R-lower	2	11	Alive
14	7P	98.6	86.1	112	103	Transient macroscopic hematuria	1.3	L-central	2	14	Alive
15	6A	52.2	49.5	119	117	None	1.1	L-upper	2	12	Alive
	6P					None	1.3	L-lower	2

**Table 2:** Overall Patient Characteristics

Patient and tumor characteristics	*n* = 15 patients (*n* = 19tumors)
Tumor size (cm), median (IQR)	2.4 (1.3–2.9)
R.E.N.A.L. score, median (IQR)	6 (5.5–8)
**Tumor polarity**	
Upper pole	8
Interpolar	4
Lower pole	7
Operation time (min), median (IQR)	66 (24–69)
**Pathology**	
RCC	19 (19)
negative pathological results (%)	0 (0)
**Haemoglobin(g/l),median (IQR)**	
Preoperative	119 (118–122)
3-7D after operative	119 (113.5–127)
**eGRF (mL/min), median (IQR)**	
Preoperative	48.1 (31.9–64.35)
1-2 M after operative	46.75 (36.95–64.025)
Hospital length,median (IQR)	5 (4–7)
**Comorbidities**	
Transient gross hematuria	2
Self-limiting perinephric hematomas	1

Patients must meet the following conditions to participate in this research:

Complete medical record materials were available and the concomitant diseases were well controlled after treatment.
Routine blood count: leucocyte count≥3*10^9^/l, neutrophil count≥2*10^9^/l, hemoglobin≥90 g/l, the platelet count≥100*10^9^/l.
3.DIC: prothrombin time:(international normalized ratio, INR) ≥1.5.
Liver and kidney functions: serum total bilirubin (TBIL) < 75umol /l, direct bilirubin (DBIL) < 39umol/l.
Patients who have not the surgical indication or nor willing to accept surgical treatment.
The expecting life span≥6 months.

Exclusive criteria:

1. Patients who have severe coronary artery disease,myelosuppression, acute or chronic infectious diseases, or cannot tolerate general anesthesia.
2. Patients who have coagulation disorders.
3. The preoperative imaging examinations confirmed no distant metastasis.

Eight of the patients were men, and 7 were women, with ages ranging from 51 to 84(median of 63 (IQR 54.5–68) years). Among them, 7 patients underwent nephrectomy for contralateral renal cancer. Four patients had two lesions in the ipsilateral kidney. One patient had nephrotic syndrome. The results of percutaneous biopsy for histopathology were renal clear cell carcinoma. Seventeen of the tumor locations were peripheral, and the other two were centrally located. The selection of the therapy modality was determined by the evaluation of the clinical multidisciplinary comprehensive treatment team. The patients’ characteristics are listed in Table 1. Routine blood count,liverfunction and renal function (blood urea nitrogen, eGFR) were obtained within 3 days before IRE treatment.

### Preanesthetic preparation and anesthesia management

After a patient entered the operating room,venous access, noninvasive blood pressure, five-lead electrocardiogram and pulse oxygen saturation were established. General anesthesia was combined intravenous and inhalation anesthesia by tracheal intubation and venous access. Drugs for general anesthesia induction included fentanyl (2txg/kg),propofol (2 mg/kg), and rocuronium (0.6 mg/kg). Intravenous pumping of Diprivan (50–150 mg/h) and inhalation of sevoflurane were used for anesthesia maintenance. Then, enhanced CT scanning was performed to determine the exact size of the tumors,surrounding structures and best puncture path.

### Irreversible electroporation procedure

The Nanoknife®System (AngioDynamics, Latham, New York) for IRE was used. The electrodes’ effective exposure length was approximately 1–2 cm. Three or four electrodeswere placed under CTguidance around the boundary of the renal tumors according to the size of the tumor. Partial zonal treatment was conducted with the tumor lesion between 2.5 cm and 4.5 cm to achieve adequate,overlapping therapeutic ablation coverage. The usual treatment region covered atumor-free margin of 5 mm in every direction. All electrodes were arranged in parallel, and the distance between each electrode pair was 1.2–2.3 cm. After the electrodes were put in place,ablation was set with the synchronous ECG trigger mode. Electroporation was performed with a cardiac synchronizer to ensure pulses in the ventricular refractory period and avoid arrhythmia. The discharge pulse times of each group are 70 ~ 90 times. The pulse length was 70 ~ 90 Ixs, the number of pulses per electrode pair was 3–13, and the average voltage was 2200–3000 V. The electrodes were pulled out after ablation, and repeated enhanced CT scanning was simultaneously performed to assess adequateablationand to observehemorrhage or exudation. The patientreceived symptomatic and supported treatment while recovering in thepostanesthesia care unit.

### Follow-up

One day, 1 week, 1 month,3 months,6 months,and 12 months after IRE, routine blood count, liver,and renal functions (BUN, Scr,eGFR) were evaluated. Contrast-enhanced computed tomography imaging changes were evaluated immediately after IRE. Contrast-enhanced computed tomography/magnetic resonance was performed 1 month, 3 months, 6 months, 12 months and every year later. Sagittal and coronal images were reconstructed with thin-slice reconstruction. The effect of the treatment was evaluated according to American Urological Association (AUA) guidelines [1]. The radiologic definition of local recurrence was that a new enhancing tumor appeared again in the ablation zone after prior complete ablation. The radiologic definition of distant kidney recurrence was defined radiographically as tumors appearing in the contralateral kidney or other areas far away from the ablation zone.

### Statistical analysis

Statistical analysis for all 19 tumors in the 15 patients was applied to compare serum creatinine andeGFR before and after the IRE procedure. Analysis was accomplished using SPSS 22.0 (IBM Corp, U.S.A.) for the paired t test. *P* < 0.05 was considered statistically significant. Excel was used for descriptive statistics.

## Results

Technical success was achieved in 100% of patients. Fifteen cases (100%) were followed, and no patient was lost to follow-up, with a median follow-up time of 30 (IQR 14–34.5) months. The median tumor diameter was 2.4 (IQR 1.3–2.9) cm. No patients had severe adverse reactions (above stage 3 of the Clavien-Dindo complication classification) [3, 4]. Complications, such as injury of the ureteral collecting system, urinary fistula,uroschesis,emboli or infarcts in the kidney caused by ablation and reported in the pertinent literature, did not occur. Six patients had tolerable light pain at the puncture point. In 1 patient,immediately after the operation,a small amount of perirenal exudation was observed, which disappeared on CT imaging 1 month after the procedure. Self-limiting perinephric hematomas occurred in 1 patient and were obviously reduced 1 month later. The patient’s immediate post treatment haemoglobin was 98 g/l. After 3 days of conventional rehydration and hypervolemic treatment, the hemoglobin was 110 g/l. Two patients were found to have macroscopic haematuria, the immediate post treatment haemoglobin was 119 g/l和105g/l. After 3 days of conventional rehydration and hypervolemic treatment, the hemoglobin was rechecked as 120 g/l and 103 g/l. In addition, the patient who was found to have perinephric haematoma also got a routine inspection. Five patients presentedmicroscopichematuria 1 day after theprocedure. Transient gross hematuria occurred in 2 patients (centrally located). Without specifictreatment, the symptom of hematuria was relieved in 3 ~ 5 days.

**Fig. 1:**
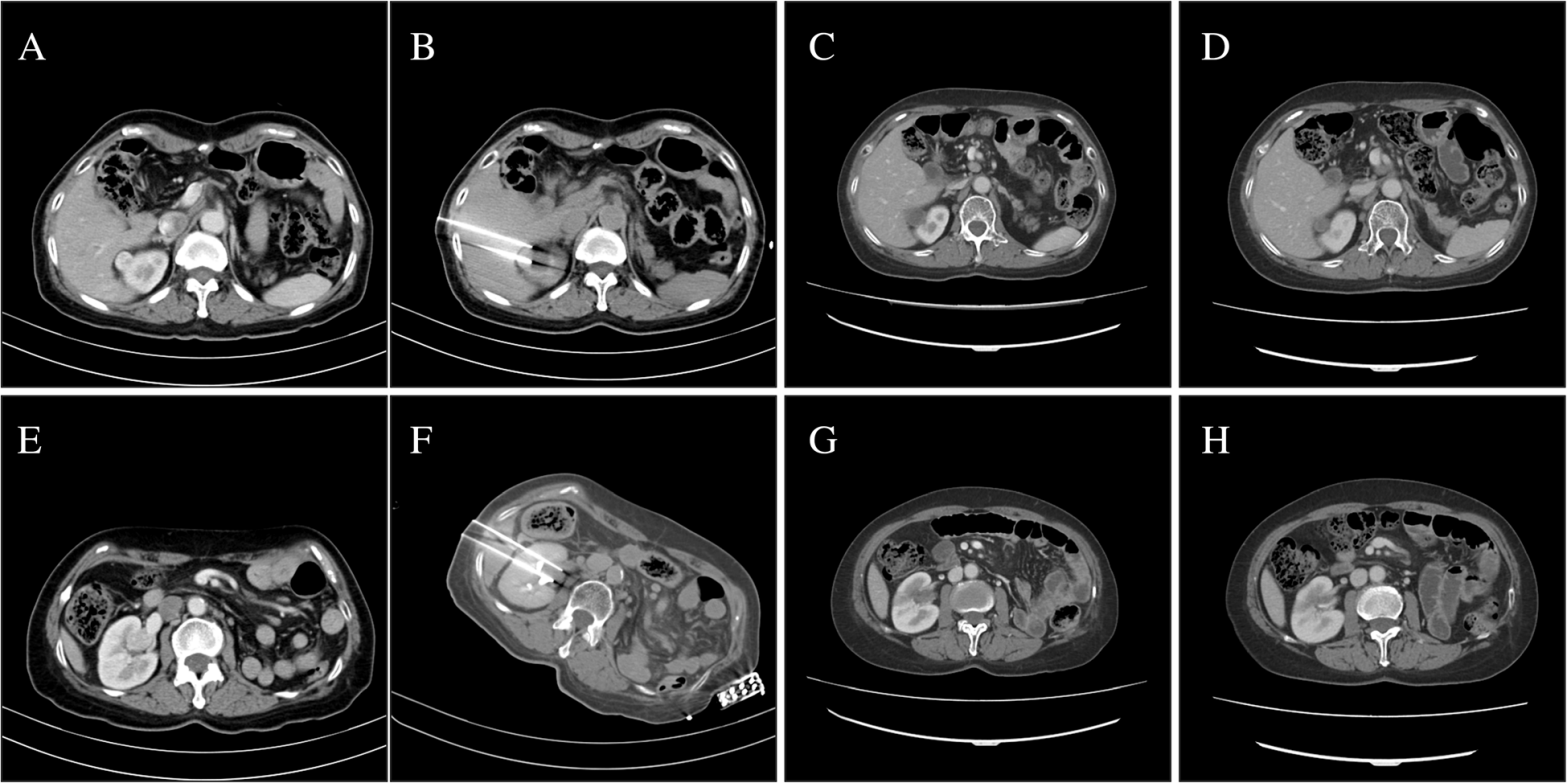
Solitary kidney patient with two RCC lesions. **a**-**d** The tumor located in the upper pole of the right kidney with the needle transhepatic approach showed no contrast enhancement 1 month (**c**) and 3 months (**d**) after IRE. **e**-**h** The tumor was adjacent to the renal pelvis with the needle transhepatic approach. Contrast-enhanced CT images obtained 1 month (**g**) and 3 months (**h**) after IRE demonstrated no enhancement in a well-circumscribed healing area without damage to the renal pelvis or large hilar vessels

**Fig. 2:**
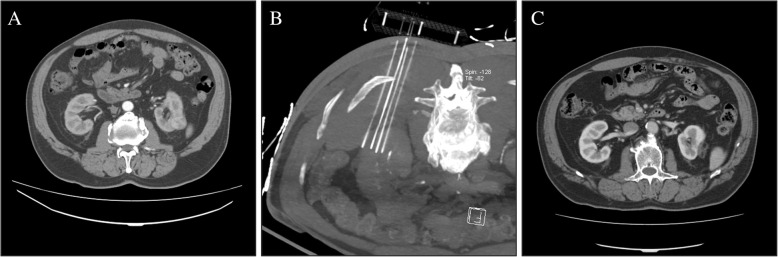
**a** Contrast-enhanced CT transversal image before IRE with the tumor located in the middle pole of the left kidney. **b** Reconstructions of the CT scan after the placement of four needles. **c** Contrast-enhanced CT axial image 18 months after IRE. The renal mass showed no contrast enhancement. The maximal diameter of the tumor was reduced compared with that in the baseline image

**Fig. 3:**
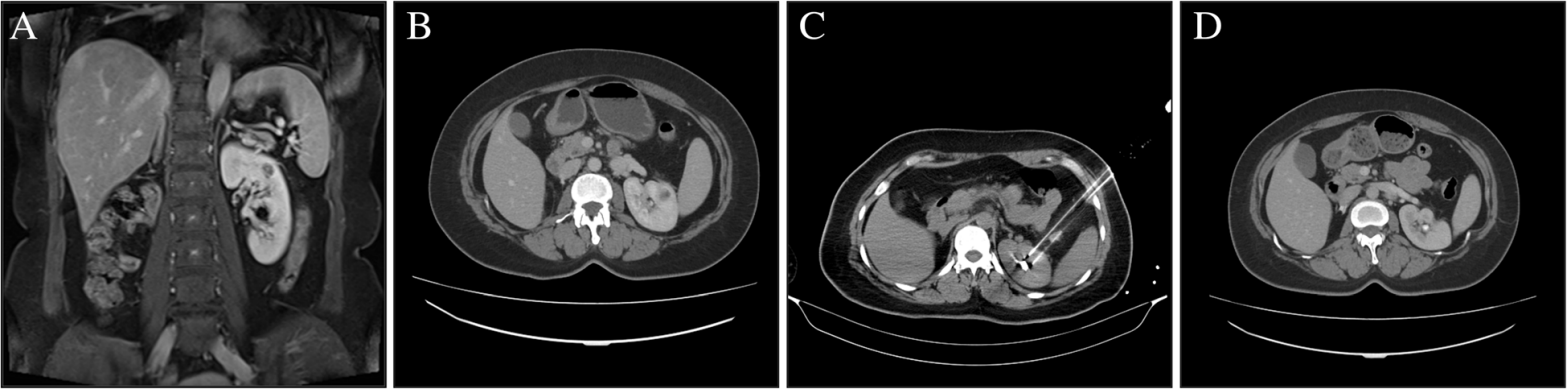
**a**-**b** Contrast-enhanced MRI coronal image and contrast-enhanced CT transversal image with the tumor located in the upper pole of the right kidney. **c** The needles were placed percutaneously through the left colon-spleen space with CT guidance. **d** Contrast-enhanced CT axial image 15 months after IRE. The kidney parenchyma atrophied, and the tum

Complete ablation was achieved in all 19 lesions in the immediately contrast-enhanced CT imaging changes,while the normal renal parenchyma vascular structure was well preserved (Figs. 1, 2, 3). The median IRE time was 66 (IQR 24–69) min. The median number of total pulses was 1706(IQR 850–1990). During thefollow-up period, complete ablation was accomplished in 14 of 15 patients (93.3%). The patients’ CT appearancesincluded low density and nonenhancement. Residual enhancement was noted in 1 of the 15 patients (6.67%) 12 months after the IRE procedure by enhancedCT scan. Subsequently, this patientaccepted salvage IRE therapy. Distant metastasis was observed in 1 patient in the needle puncture track, and the metastatic lesion was surgically removed. The other patients had no evidence of recurrence found on the follow-upimaging. There were no significant differences in the Scr before and after (last follow-up) the IRE procedure(*P* > 0.05), even in one patient withrenal dysfunction.

## Discussion

Current guidelines consider thermal ablation as an alternative to nephron-sparing surgery (NSS) for patients who have renal dysfunction or have no surgical indication [6]. Recently, the long-term efficacy of ablative therapy has been reported to be comparable to the outcome of surgical resection, with the greatest retention of renal function and few major complications. Ablative therapy is a minimally invasive treatment that maximizes nephron retention and is usually appropriate for patients who are ineligible for surgical resection, have only a solitary functioning kidney or have combined renal dysfunction [7]. By using electric pulses to create nanopores in the cell membrane of tumor cells, IRE will eventually induce cell apoptosis. Since the scope of IRE is limited to the cell membrane, the other extracellular matrix structuresare not affected, which allows the rapid regeneration of normal tissues.

Compared with the decrease in renal function after thermal ablation for RCC [8], no significant changes in renal function or serious complications ofIRE treatment forRCC have been reported in the literature. Noah E. Canvasser et al. [9] chose 42 cT1a RCC lesions from 41 patients with IRE. The initial treatment success rate was 93%(39/42), and the 2-year local-recurrence-free survival (LRFS) was 83%.No major complications occurred. Steffen J. Diehl et al. [10] described a series of 5 patients with 7 potentially malignant renal lesions in a solitary kidney treated with IRE. The author reported no significant decrease in eGFR over 3 months, even though 1 patient’s eGFR decreased from >60 ml/min/1.73 m2 to 44 ml/min/1.73 m2. In our study,there were no significant changes in serum creatinine, serum urea nitrogen and eGFR after IRE therapy compared with the corresponding values before IRE in 15 patients, including 1 patient with nephrotic syndrome and 7 patients with a solitary kidney. Moreover, the normal renal function was completely preserved. No serious complications (Clavien-Dindo grade III or above) occurred.

The presence of a “heat sink effect” in thermal ablation, such as RFA and MWA,can affect the outcome of thermal ablation in lesions close to the vessel. There is also a “warming effect” of cryoablation [10, 11]. The incidence of postoperative hemorrhage, intraoperative area infection, urine leakage, ureteral injury and stricture, intestinal damage and other nontherapeutic target organs caused by ablation is relatively high (4%) [12, 13]. However, IRE does not have these limitations. For centrally located kidney tumors, IRE has advantages over surgical resection and thermal ablation techniques. At the same time, compared with thermal ablation and cryoablation, IRE requires no preoperative preparation for malignant renal tumors near important organs and tissues (such as pyelostomy, ureteral stent implantation, and artificial pneumoperitoneum and ascites, etc., to protect important tissues and organs). Transient gross hematuria occurred in 2 patients with centrally located lesions without any otherserious postoperative complications inour study.

The tumor maximal diameter of patient 10 was 4.5 cm. IRE was not recommended for this situation because the diameter exceeded the surgical indications. The guidelines also do not recommend ablation for tumors larger than 3 cm in diameter. At the same time, the related literature also pointed out that the risk of recurrence is higher [1, 14]. The patient 10 chose to try the IRE treatment and had a satisfactory curative effect. Regarding the complications and deficiencies caused by IRE, the latest literature has been explored in animal experiments to find improved methods. Timothy J. O’Brien et al. [15] tested the effects of using several pulse-timing paradigms on electrical current, tissue temperature, and tissue treatment size. They believe that cycled pulsing patterns may hold promise for enhancing the efficacy of IRE application in clinical practice and could lead to better overall outcomes for patients. N. Beitel-White et al. [16] found that larger overall changes in output current are correlated with larger decreases in T cell populations 24 h after treatment. Real-time decisions can be made regarding the optimal follow-up therapy based on the range of output current delivered during treatment. This approach will also maximize the immunomodulating effect of IRE in synergy with follow-up immunotherapy.

Safe and successful CT-guided percutaneous ablation of RCC by IRE begins with careful preprocedural evaluation of imaging to plan the appropriate patient position, needle approach, and trajectory. The kidney is surrounded by the liver, diaphragm, lower lobe of the lung, vertebral bodies, spleen, and bowel. Given the anatomical relationship of the kidney and its surrounding organs, a suitable approach should be used according to the location of the tumor to avoid major complications related to the puncture procedure, such as scarring with ureteropelvic junction obstruction, neuromuscular injury, and bowel wall perforation.

It has been reported in the relevant literature that thermal ablation of renal tumors via the anterior approach is considered to be a relative contraindication. The main reason is the risk of percutaneous ablation-related complications, such as damage to nontarget organs, including the small intestine, pancreas, colon,etc. [17, 18]. Hegg et al. [19] reported 18 patients (19 tumors) who underwent transhepatic renal RFA procedures by ultrasound guidance without the development of major complications during the procedures. Ginat et al. [20] described several bowel displacement and protection techniques during percutaneous renal tumor thermal ablation to avoid potential devastating consequences. In our treatment experience, CT guidance is the more suitable method for the IRE procedure because of its ability to reveal the detailed anatomy along the electrodeneedle pathway. The transhepatic approach is safe forthe treatment of tumors located anterolaterally in the right kidney. The left colon-spleen space is appropriate for tumors located at the anterior border of the left kidney. Most tumors located in the lateral and posterior part of the kidney are suitable for a posterior approach. Combined with other literatures and our experience, we can explain the ideal patient, candidate to IRE. First, it is reserved for patients who have not the surgical indication, or are prone to have postoperative complications and the patient who are not willing to accept surgical treatment. Second, IRE is suitable for the patients with higher risk of postoperative complications after thermal ablation (patients with tumor close to the urinary collection system, vital organs and great vessels, or with high R.E.N.A.L. score). Third, patients with renal insufficiency or patient who has a solitary kidney are also the ideal candidate for IRE treatment.

The current study is limited by its retrospective nature. There was no comparison arm of patients. The patient sample size wassmall, and the follow-up time was relatively short. In recent years, IRE technology for RCC has remained relatively less developed globally,and thus, the safety and efficacy of this treatment need to be further clarified through a prospective study design with a larger sample size.

## Conclusions

According to the initial study results, percutaneous treatment of renal malignant tumors with IRE appears to be a safe, effective and feasibletherapy for RCC. The IRE technique has demonstrated a good therapeutic effect and protects renal function in patients without surgical indications,even with centrally located lesions. The present study may help to better plan appropriate patient positions, needle approaches, and trajectories.

## Data Availability

The datasets used and/or analysed during the current study are available from the corresponding author on reasonable request.

## Abbreviations

IRE: Irreversible electroporation
RCC: Renal cell carcinoma
eGFR: Estimated glomerular filtration rate
RFA: Radiofrequency ablation
EUA: European association of urology
MWA: Microwave ablation
INR: International normalized ratio
TBIL: Total bilirubin
DBIL: Direct bilirubin
BUN: Blood urea nitrogen
Scr: Serum creatinine
AUA: American urological association
NSS: Nephron-sparing surgery
LRFS: Local-recurrence-free survival
